# Clinicopathological characteristics and survival of spinal cord astrocytomas

**DOI:** 10.1002/cam4.3364

**Published:** 2020-08-10

**Authors:** Yao‐Wu Zhang, Rui‐Chao Chai, Ren Cao, Wen‐Ju Jiang, Wei‐Hao Liu, Yu‐Lun Xu, Jun Yang, Yong‐Zhi Wang, Wen‐Qing Jia

**Affiliations:** ^1^ Department of Neurosurgery Beijing Tiantan Hospital, Capital Medical University Beijing China; ^2^ China National Clinical Research Center for Neurological Diseases Beijing China; ^3^ Department of Molecular Neuropathology Beijing neurosurgical institute, Capital Medical University Beijing China; ^4^ Chinese Glioma Genome Atlas Network (CGGA) Beijing China

**Keywords:** astrocytoma, clinicopathological feature, H3 K27M, spinal cord tumor, survival

## Abstract

**Background:**

Due to their rarity, the clinicopathological characteristics and prognostic factors of spinal cord gliomas are still unclear. Here, we aimed to clarify these issues in a cohort of 108 spinal cord astrocytomas.

**Methods:**

We characterized the clinicopathological characteristics, including 2016 World Health Organization (WHO) grade, age, sex, location, segment length, resection, pre‐ and postsurgery, Modified McCormick Scale (MMS), radio‐ and chemotherapy, and Ki‐67 and H3 K27M mutations, in 108 spinal cord astrocytomas through heatmaps. The Cox regression analysis and Kaplan‐Meier curves were used to study the prognostic value of these clinicopathological features.

**Results:**

There are a total 38 H3 K27M‐mutant tumors, including 31 cases with histological grade II/III tumors. The age of low‐grade astrocytoma patients (WHO grade I/II, n = 54) was significantly younger (27.0 vs 35.5 years, *P* = .001) than those with high‐grade tumors (WHO grade III/IV, n = 54). All patients underwent surgical resection with neurophysiological monitoring, and the surgery did not result in significant changes in MMS. The presurgery MMS was associated with overall survival in the high‐grade subgroup (*P* = .008) but not in the low‐grade subgroup (*P* = .312). While, the high content of resection improved the survival of only patients with low‐grade astrocytomas (*P* = .016) but not those with high‐grade astrocytomas (*P* = .475). Both the low‐grade and high‐grade astrocytomas had no obvious benefit from neither adjuvant chemotherapy nor radiotherapy (all *P* > .05).

**Conclusions:**

We characterized the clinicopathological characteristics and their prognostic values in 108 spinal cord astrocytomas, which could help with evidence‐based management of spinal cord astrocytomas.

## Graphical abstract

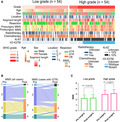

## 1 INTRODUCTION

Spinal cord gliomas (including those of the cauda equina) are relatively uncommon neoplasms, accounting for approximately 4.2% of all central nervous system (CNS) gliomas, of which 20%‐40% are astrocytomas and the rest are ependymomas.^1^ Although treatment guidelines for brain gliomas, prognostic molecular markers, and prognostic markers associated with chemo‐ and radiotherapy have been established, spinal cord gliomas are considerably different from brain gliomas in terms of biological behaviors and molecular profiles.^2^, ^3^, ^4^, ^5^, ^6^, ^7^, ^8^ Special treatment guidelines are yet to be established for spinal cord gliomas because of their relatively rare incidence, and the characteristics and prognostic factors of spinal cord astrocytomas remain largely unknown.

Previous studies have investigated the prognostic factors of spinal cord astrocytomas.^9^, ^10^, ^11^, ^12^, ^13^, ^14^, ^15^ However, most of these studies are small samples or multicenter, and the findings are inconsistent, such as the effect of surgical extent on the prognosis of low‐grade or high‐grade spinal cord astrocytomas.^16^ The schemes and effects of adjuvant radiotherapy or chemotherapy vary from institution to institution.^9^, ^16^, ^17^, ^18^

“Diffused midline glioma, H3 K27M‐mutant” has been defined as a new entity in the 2016 World Health Organization (WHO) classification of Central Nervous System (CNS) tumors, corresponding to WHO grade IV.^19^ It mainly occurs in the midline structure of the thalamus, brainstem, and spinal cord. Some studies have reported “diffuse midline glioma, H3 K27M‐mutant” in the midline structure including spinal cord.^17^, ^20^, ^21^, ^22^ However, based on the 2016 WHO classification, the clinicopathological characteristics of spinal cord astrocytomas are rarely reported. It remains challenging to manage spinal cord astrocytomas with evidence‐based practices. Therefore, it is urgent to summarize the characteristics and prognostic factors of spinal cord astrocytomas from a relatively large number of patients based on the 2016 WHO classification.

This study aimed to determine the clinical characteristics and prognostic factors of spinal cord astrocytomas based on the 2016 WHO classification through a retrospective analysis of 108 patients. Our findings will provide information on the overall median survival (OS) of patients with spinal cord astrocytomas of different WHO grades. We also compared the clinicopathological characteristics and prognostic impacts of interventions between low‐grade (WHO grade I/II) and high‐grade (WHO grade III/IV) gliomas.

## 2 MATERIALS AND METHODS

### 2.1 Patient population

We reviewed 108 patients who underwent open surgical resection and who were pathologically confirmed to have spinal cord astrocytomas at our hospital from 2011 to 2018. Among all patients, three were excluded from the final survival analysis because of loss to follow‐up (n = 2) or death during the perioperative period (n = 1). The patient demographics, including age, sex, tumor location, histology, radiotherapy, and chemotherapy, are shown in Table 1.

**TABLE 1:** Characteristics of patients with spinal cord astrocytomas who were enrolled in the study

		All patients (N = 108)	Low‐grade (N = 54)	High‐grade (N = 54)	*P*‐value^a^
Variable		N (%)	N (%)	N (%)	
Age (y)	Median (range)	30.0 (4‐63)	27.0 (4‐54)	35.5 (8‐63)	**.001**
Sex	Female	41 (38.0%)	16 (29.6%)	25 (46.3%)	.074
	Male	67 (62.0%)	38 (70.4%)	29 (53.7%)	
Location	C	40 (37.0%)	21 (38.9%)	19 (35.2%)	.896
	C‐T	16 (14.8%)	8 (14.8%)	8 (14.8%)	
	T	44 (40.8%)	22 (40.7%)	22 (40.7%)	
	T‐L	8 (7.4%)	3 (5.6%)	5 (9.3%)	
Segment length	Average	3.91	3.78	4.04	.434
Resection	GTR	58 (53.7%)	35 (64.8%)	23 (42.6%)	**.054**
	STR	18 (16.7%)	8 (14.8%)	10 (18.5%)	
	OB	32 (29.6%)	11 (20.4%)	21 (38.9%)	
Presurgery MMS	1	4 (3.7%)	3 (5.7%)	1 (1.9%)	**<.001**
	2	50 (46.7%)	37 (69.8%)	13 (24.1%)	
	3	36 (33.6%)	10 (18.9%)	26 (48.1%)	
	4	17 (15.9%)	3 (5.7%)	14 (25.9%)	
	NA	1	1	0	
Postsurgery MMS	1	5 (4.7%)	4 (7.5%)	1 (1.9%)	**<.001**
	2	39 (36.4%)	31 (58.5%)	8 (14.8%)	
	3	44 (41.1%)	16 (30.2%)	28 (51.9%)	
	4	19 (17.8%)	2 (3.8%)	17 (31.5%)	
	NA	1	1	0	
Radiotherapy	Yes	62 (67.4%)	32 (66.7%)	30 (68.2%)	.877
	No	30 (32.6%)	16 (33.3%)	14 (31.8%)	
	NA	16	6	10	
Chemotherapy	Yes	27 (29.3%)	8 (16.7%)	19 (43.2%)	**.005**
	No	65 (70.7%)	40 (83.3%)	25 (56.8%)	
	NA	16	6	10	
Ki‐67	<10%	53 (51.0%)	42 (82.4%)	11 (20.8%)	**<.001**
	≥10%	51 (49.0%)	9 (19.6%)	42 (79.2%)	
	NA	4	3	1	
H3 K27M	Negative	62 (62.0%)	—	15 (28.3%)	—
	Positive	38 (38.0%)	—	38 (71.7%)	
	NA	8^b^	7	1	

*Note:* Significance of bold values *P* < .05. Abbreviations: C, cervical; C‐T, cervicothoracic; GTR, gross total resection; MMS, Modified McCormick Scale; NA, not available; OB, open biopsy; STR, subtotal resection; T, thoracic; T‐L, thoracolumbar.

^a^ *P*‐values for the *t* test or Chi‐squared test or Mann‐Whitney U test used to compare low‐grade and high‐grade tumors.

^b^ Patients before 2016 without data on H3 K27M mutation and without adequate tissue sample available for added immunohistochemistry, including 2, 5, and 1 patient with grade I, II, and III tumors, respectively.

### 2.2 Neuropathologic evaluation

Routine neuropathologic evaluation of the formalin‐fixed and paraffin‐embedded tumor samples included hematoxylin and eosin staining and immunohistochemical analysis performed using antibodies against mutant H3 K27M (ABE419; Millipore; 1:800) and Ki‐67 (MIB‐1; Labvision; 1:50). K27M‐mutant gliomas were classified as grade IV according to the 2016 WHO Classification of Tumors of the Central Nervous System.^19^

### 2.3 Treatment modality

The posterior midline approach with the lateral oblique side of the lesion in the upper position was adopted for all cases, and laminectomy was customized for solid tumors. We routinely used sensory and motor evoked potentials in microsurgical resection. While monitoring the changes in sensory and motor evoked potentials, we attempted to perform maximal resection of the tumor according to the extent allowed by the spinal cord function. If the presence of sensory or motor evoked potentials sustained decrease >50% of the baseline or the surgical plane could not be identified, dissection was stopped, and gentle manipulation was considered during the whole procedure. If the spinal cord is severely swollen before surgery, laminectomy is usually performed for decompression. Surgical interventions and subsequent medical management were performed by the same group of surgeons, following identical procedures. Neurological functional status was retrospectively evaluated using the Modified McCormick Scale (MMS).^23^

The extent of resection (EOR) was estimated using postsurgery magnetic resonance images (MRI). Based on the MRI results, EOR was defined as gross total resection (≥90%), subtotal resection (≥50% and <90%),^17^ and open biopsy (<50%). The EOR was independently determined by two experienced radiologists, who were blinded to the clinical data of the patients. In case of a discrepancy, the two observers simultaneously reviewed the images to achieve a consensus.

Owing to the lack of standard chemo‐ and radiotherapy regimens for spinal cord gliomas, the chemoradiotherapy regimen for spinal cord gliomas is based on the treatment protocol for brain gliomas, according to the National Comprehensive Cancer Network (NCCN) guidelines. The postsurgery conventional adjuvant radiotherapy total dose was 40‐50 Gy, which was administered at a dose of 1.8‐2.0 Gy per fraction per day for 5 days per treatment week over 5 consecutive weeks (23‐25 days). The chemotherapy regimen adopted temozolomide, administered at a daily dose of 75 mg/m^2^ during radiotherapy or at a daily dose of 150/200 mg/m^2^ for 5 consecutive days per treatment week over 4 weeks per cycle.

### 2.4 Statistical analysis

The characteristics of patients and lesions are presented by descriptive statistics. Differences in age were analyzed using the t‐test, and categorical variables were analyzed using the Chi‐squared test or Mann‐Whitney U test. OS was defined as the period from the date of surgery to the date of last follow‐up or death. Prognostic factors and survival were analyzed, and the results were compared using the Kaplan‐Meier and log‐rank tests and univariate and multivariate Cox regression. Hazard ratios (HRs) with 95% confidence intervals (CIs) were calculated. Survival rates were calculated at 1 year, 3 years, 5 years, and median survival times (standard error, 95% CI). All *P*‐values < .05 were considered statistically significant, and statistical analyses were conducted using IBM SPSS Statistics software (Release 23.0.0) and GraphPad Prism 7 (GraphPad Software).

## 3 RESULTS

### 3.1 Baseline demographics

**FIGURE 1:**
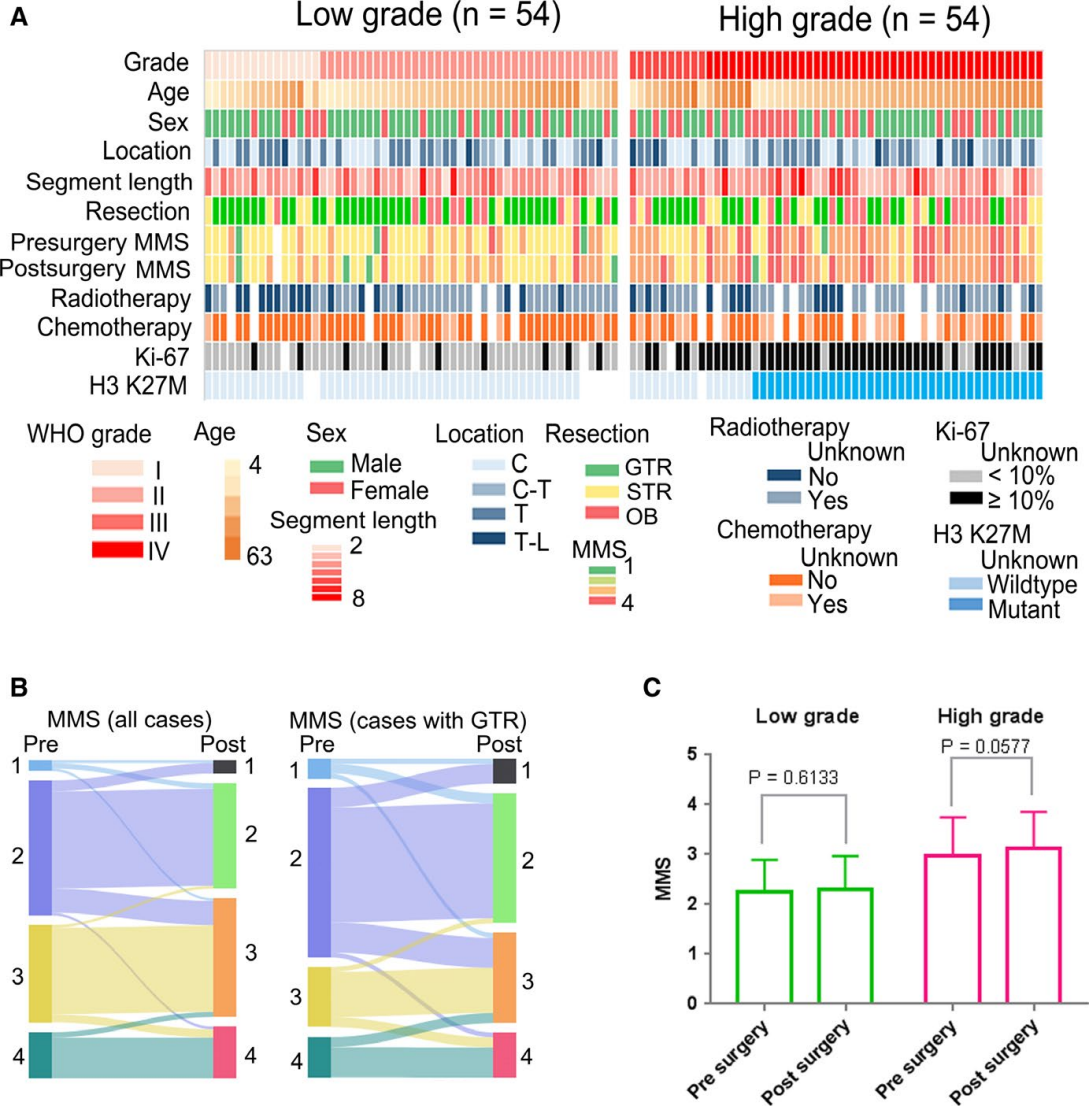
(A) A map of the characteristics of patients enrolled in this study. Age in years. (B) Presurgery and postsurgery changes in MMS in all patients and GTR patients. (C) Changes in MMS for presurgery and postsurgery low‐grade and high‐grade tumors. C, cervical; C‐T, cervicothoracic; GTR, gross total resection; MMS, Modified McCormick Scale; OB, open biopsy; STR, subtotal resection; T, thoracic; T‐L, thoracolumbar

The characteristics of the patients are shown in Figure 1A and Table 1. The male‐to‐female ratio was 1.6:1. The median age at the time of diagnosis was 30.0 years (range, 4‐63 years). Patients with low‐grade astrocytomas were younger than those with high‐grade tumors (median age, 27.0 vs 35.5 years; *P* = .001). The thoracic vertebrae (40.8%) were the most common tumor locations, followed by the cervical (37.0%), cervicothoracic (14.8%), and thoracolumbar (7.4%) vertebrae. There was no significant correlation between pathological grading and tumor location (*P* = .896). The average tumor span was 3.91 segments, and the tumor span between low‐ and high‐grade astrocytomas showed no significant difference (*P* = .434).

All patients underwent open surgical resection with neurophysiological monitoring. Fifty‐eight patients (53.7%) underwent gross total resection, 18 (16.7%) underwent subtotal resection, and the rest (29.6%) had open biopsy. A remarkable difference in EOR was found between low‐grade and high‐grade astrocytomas, and the open biopsy ratio was increased in high‐grade astrocytomas (38.9% vs 20.4%, *P* = .054, Table 1). Among patients with low‐grade astrocytomas, 75.5% had a presurgery MMS of grade 1 or 2, while 74.0% of those with high‐grade astrocytomas had a presurgery MMS of grade 3 or 4. The presurgery MMS of patients with high‐grade tumor was significantly higher than those of patients with low‐grade tumor (*P* < .001). Presurgery and postsurgery changes in MMS are shown in Figure 1B. The surgery did not significantly change the MMS in patients with low‐grade tumor (*P* = .613), it slightly increased the MMS in patients with high‐grade tumor, but the increase was not insignificant (*P* = .058, Figure 1C). Information on postsurgery adjuvant chemo‐ and radiotherapy was available in 92 patients (85.2%). Among them, 66.7% of those with low‐grade tumors and 68.2% of those with high‐grade tumors received adjuvant radiotherapy (*P* = .877). A higher ratio of patients with high‐grade tumors (43.2% vs 16.7%, *P* = .005) received chemotherapy.

In this study, the positive rate of H3 K27M mutation was 38%, and 31 histological grade II/III gliomas with H3 K27M mutation were diagnosed as WHO grade IV gliomas. Grade I, II, III, and IV gliomas were diagnosed in 15 (13.9%), 39 (36.1%), 10 (9.3%), and 44 (40.7%) patients, respectively. Ki‐67 showed a high expression rate (≥10%) in almost 80% of cases of high‐grade astrocytomas (WHO grade III/IV), which was remarkably higher (*P* < .001) than in cases of low‐grade tumors (WHO grade I/II).

### 3.2 OS in low‐grade and high‐grade astrocytomas

**FIGURE 2:**
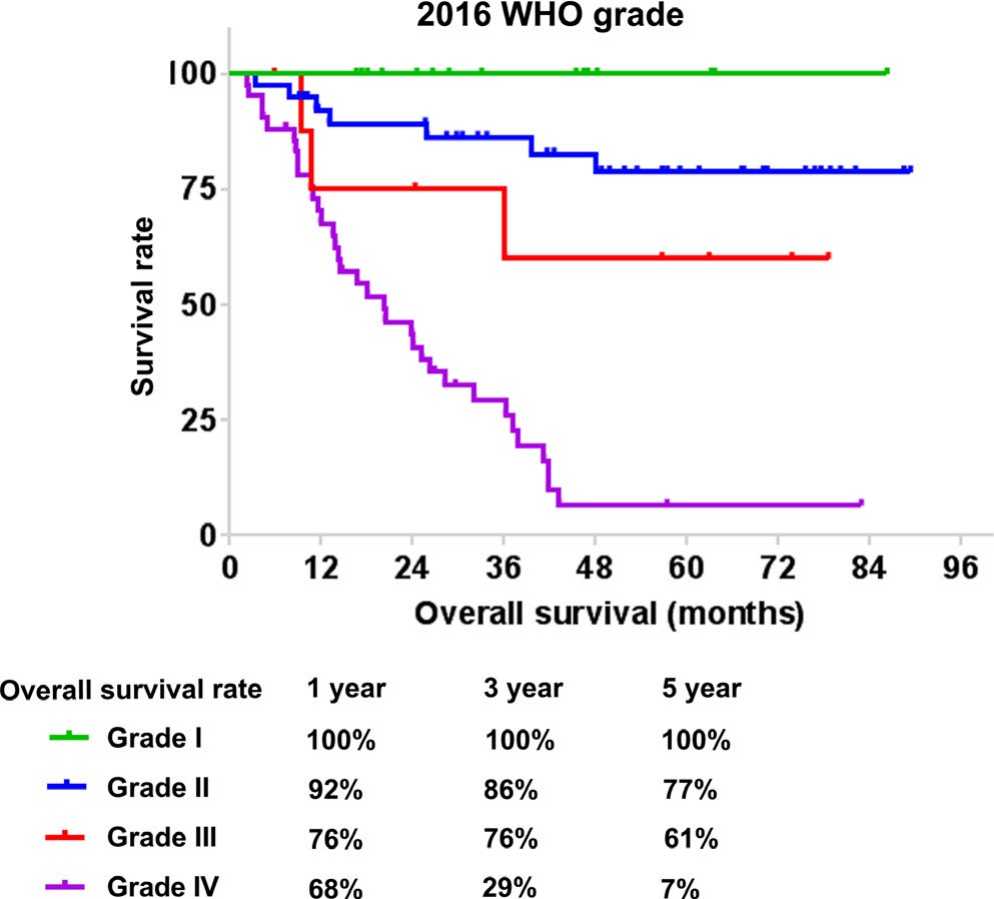
Kaplan‐Meier survival analysis of survival rate in patients with astrocytoma based on the 2016 WHO classification

Follow‐up data were available in 106 patients (98.0%). The median follow‐up time was 50.2 months after surgery, and 45 patients died during the period. The Kaplan‐Meier estimates of OS according to WHO grades are shown in Figure 2. The median OS time of patients with grade IV tumors was 20.2 months, while that of patients with grade I, II, or III tumors was not reached. All patients with grade I tumors survived until the last follow‐up. Meanwhile, the 1‐, 3‐, and 5‐year OS rates were 92%, 86%, and 77% for grade II tumors, 76%, 76%, and 61% for grade III tumors, and only 68%, 29%, and 7% for grade IV tumors, respectively.

### 3.3 Prognostic factors of low‐grade and high‐grade astrocytomas

**FIGURE 3:**
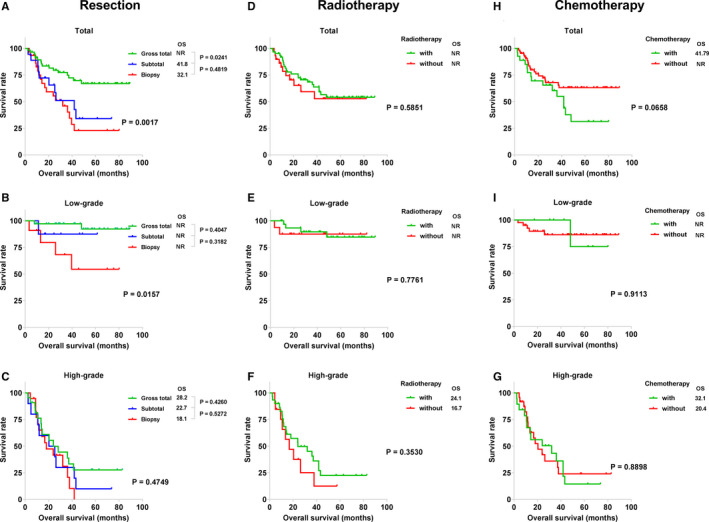
Kaplan‐Meier survival analysis of present treatment, showing the effects of the extent of resection (A, total; B, low‐grade; C, high‐grade), radiotherapy (D, total; E, low‐grade; F, high‐grade), and chemotherapy (H, total; I, low‐grade; G, high‐grade) on patient survival. NR, not reached; OS, overall median survival

Univariate Cox regression analyses of clinicopathological factors are shown in Table 2. For all patient groups, older age (HR, 1.03; 95% CI, 1.01‐1.05; *P* = .007), higher WHO grade (HR, 3.33; 95% CI, 2.24‐4.96; *P* < .001), lower EOR (HR, 1.78; 95% CI, 1.28‐2.47; *P* = .001), higher presurgery MMS (HR, 2.77; 95% CI, 1.87‐4.10; *P* < .001), and Ki‐67 expression rate ≥10% (HR, 6.33; 95% CI, 3.09‐12.93; *P* < .001) were significantly associated with worse survival. For low‐grade gliomas, EOR was significantly correlated with OS (HR, 2.89; 95% CI, 1.24‐6.74; *P* = .014). For high‐grade gliomas, WHO grade (HR, 4.06; 95% CI, 1.23‐13.42; *P* = .021), presurgery MMS (HR, 1.99; 95% CI, 1.20‐3.31; *P* = .008), and Ki‐67 expression (HR, 3.47; 95% CI, 1.33‐9.02; *P* = .011) were significantly correlated with OS. The Kaplan‐Meier curves also indicated that the EOR could significantly stratify the OS of all astrocytoma patients (*P* = .0017, Figure 3A) or the low‐grade astrocytoma subgroup (*P* = .0157, Figure 3B), but not the OS of the high‐grade astrocytoma subgroup (*P* = .4749, Figure 3C). It was noteworthy that both adjuvant radiotherapy and chemotherapy did not have a significant effect on median survival time for both low‐grade and high‐grade tumors (Figure 3D‐G).

**TABLE 2:** Univariate analysis of the clinicopathological features and overall survival of patients with spinal cord astrocytomas

Variable	All patients		Low‐grade		High‐grade	
	Hazard ratio (95% CI)	*P*	Hazard ratio (95% CI)	*P*	Hazard ratio (95% CI)	*P*‐value
Age (y)	1.03 (1.01‐1.05)	**.007**	1.03 (0.97‐1.09)	.323	1.00 (0.98‐1.03)	.763
Sex (male)	0.60 (0.33‐1.08)	.089	1.04 (0.20‐5.39)	.959	0.70 (0.37‐1.33)	.275
WHO grade (I‐IV)	3.33 (2.24‐4.96)	**<.001**	33.04 (0.03‐44265.88)	.341	4.06 (1.23‐13.42)	**.021**
Location (C, C‐T, T, T‐L)	0.92 (0.70‐1.22)	.574	1.00 (0.49‐2.04)	.989	0.86 (0.64‐1.16)	.325
Segment length	1.13 (0.96‐1.34)	.152	0.87 (0.53‐1.43)	.586	1.19 (0.99‐1.44)	.066
Presurgery MMS (1‐4)	2.77 (1.87‐4.10)	**<.001**	1.70 (0.61‐4.79)	.312	1.99 (1.20‐3.31)	**.008**
Resection (GTR, STR, PR)	1.78 (1.28‐2.47)	**.001**	2.89 (1.24‐6.74)	**.014**	1.26 (0.86‐1.83)	.231
Radiotherapy	0.82 (0.40‐1.68)	.586	0.78 (0.14‐4.32)	.777	0.69 (0.31‐1.53)	.357
Chemotherapy	1.86 (0.95‐3.63)	.070	0.89 (0.10‐7.59)	.911	1.05 (0.51‐2.19)	.890
Ki‐67 expression (≥10%)	6.33 (3.09‐12.93)	**<.001**	1.90 (0.37‐9.79)	.445	3.47 (1.33‐9.02)	**.011**

Note

Significance of bold values *P* < .05.

Abbreviations: C, cervical; CI, confidential interval; C‐T, cervicothoracic; GTR, gross total resection; MMS, Modified McCormick Scale; PR, partial resection or open biopsy; STR, subtotal resection; T, thoracic; T‐L, thoracolumbar.

We also performed a multivariate Cox regression analysis of factors with *P*‐values < .05 in the univariate analysis for all astrocytoma patients or the high‐grade astrocytoma subgroup, including age, WHO grade, presurgery MMS, resection, and Ki‐67 expression (Table 3). Among all patients, WHO grade (HR, 2.08; 95% CI, 1.29‐3.37; *P* = .003), presurgery MMS (HR, 1.69; 95% CI, 1.02‐2.77; *P* = .040), and Ki‐67 expression (HR, 2.43; 95% CI, 1.09‐5.40; *P* = .029) were the independent prognostic factors for OS. In the high‐grade tumor group, presurgery MMS (HR, 1.86; 95% CI, 1.06‐3.27; *P* = .031) and Ki‐67 expression (HR, 2.80; 95% CI, 1.00‐7.83; *P* = .049) were the independent prognostic factors for survival.

**TABLE 3:** Multivariate analysis of the clinicopathological features of all patients and the high‐grade spinal cord astrocytoma subgroup

Variable	All patients (N = 108)		High‐grade (N = 54)	
	Hazard ratio (95% CI)	*P*‐value	Hazard ratio (95% CI)	*P*‐value
Age (y)	1.01 (0.98‐1.03)	.656	NT	—
WHO grade (I‐IV)	2.08 (1.29‐3.37)	**.003**	2.42 (0.70‐8.35)	.163
Presurgery MMS (1‐4)	1.69 (1.02‐2.77)	**.040**	1.86 (1.06‐3.27)	**.031**
Resection (GTR, STR, PR)	1.25 (0.87‐1.79)	.227	NT	—
Ki‐67 expression (≥10%)	2.43 (1.09‐5.40)	**.029**	2.85 (1.07‐7.55)	**.036**

Note

Significance of bold values *P* < .05.

Abbreviations: CI, confidential interval; GTR, gross total resection; MMS, Modified McCormick Scale; NT, not tested;PR, partial resection or open biopsy; STR, subtotal resection.

### 3.4 Illustrative cases

#### 3.4.1 Case 1

**FIGURE 4:**
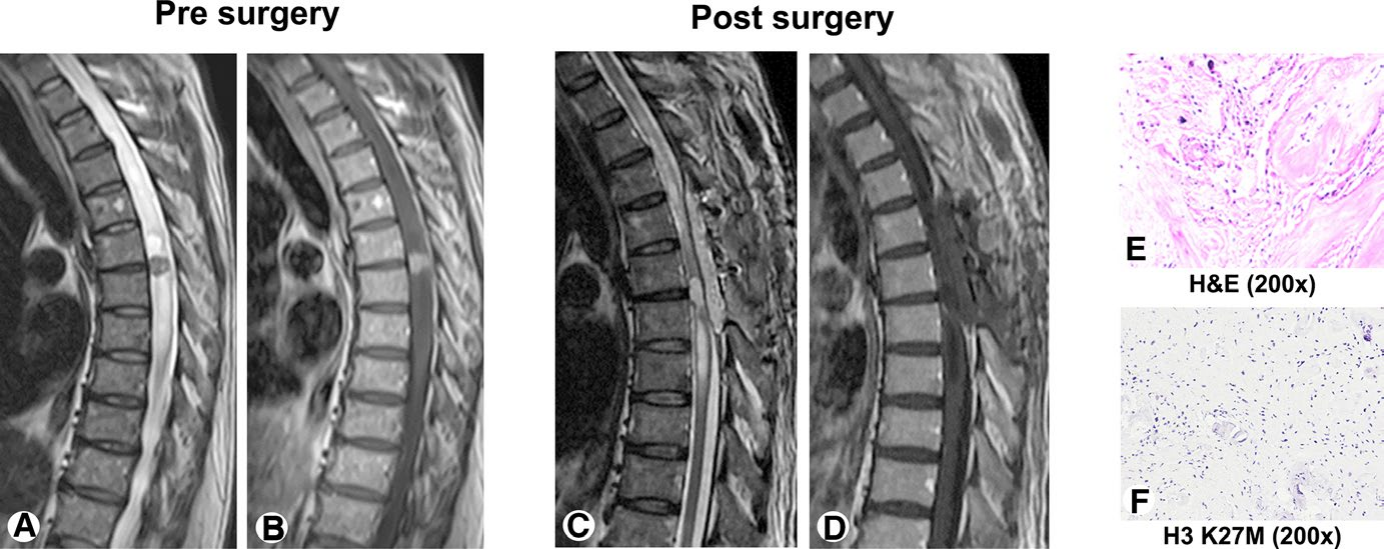
Case 1: Images of an H3 K27M mutation‐negative tumor in a 48‐year‐old male whose cardinal symptoms were numbness and weakness in the right lower limb. The histopathology identified a diffuse astrocytoma (WHO grade II). (A, B) Presurgery T2‐weighted and enhanced T1‐weighted magnetic resonance images (MRI). (C, D) Postsurgery T2‐weighted and enhanced T1‐weighted MRI. (E) Diffuse astrocytoma (H&E, 200×). (F) H3 K27M mutation‐negative tumor (H3 K27M, 200×). H&E, hematoxylin and eosin staining

A 48‐year‐old male presented with numbness and weakness of right lower limb. Presurgery neurological assessment showed grade IV muscle strength in the right lower limb. The grade of presurgery MMS was 2. Presurgery T2‐weighted MRI revealed slightly swollen thoracic cord at the T2‐T7 level, and enhanced T1‐weighted MRI showed thoracic cord nodular‐like enhancement at the T5‐T6 level (Figure 4A,B). T2‐weighted and enhanced T1‐weighted images 1 week after surgery are shown in Figure 4C,D. He underwent gross total resection, and the histopathology identified a diffuse astrocytoma (WHO grade II, Figure 4E). The H3 K27M analysis was negative (Figure 4F), and the percentage of Ki‐67‐positive cells was 3%‐5%. Postsurgery neurological assessment showed grade IV muscle strength in both lower limbs. He received adjuvant radiotherapy after surgery. At the last follow‐up (56.8 months), he demonstrated grade III muscle strength of the lower limbs and grade IV muscle strength of the upper limbs. This observation was consistent with our finding that lower WHO grade, lower presurgery MMS, higher EOR, and Ki‐67 expression rate <10% were significantly associated with better survival in low‐grade gliomas.

#### 3.4.2 Case 2

**FIGURE 5:**
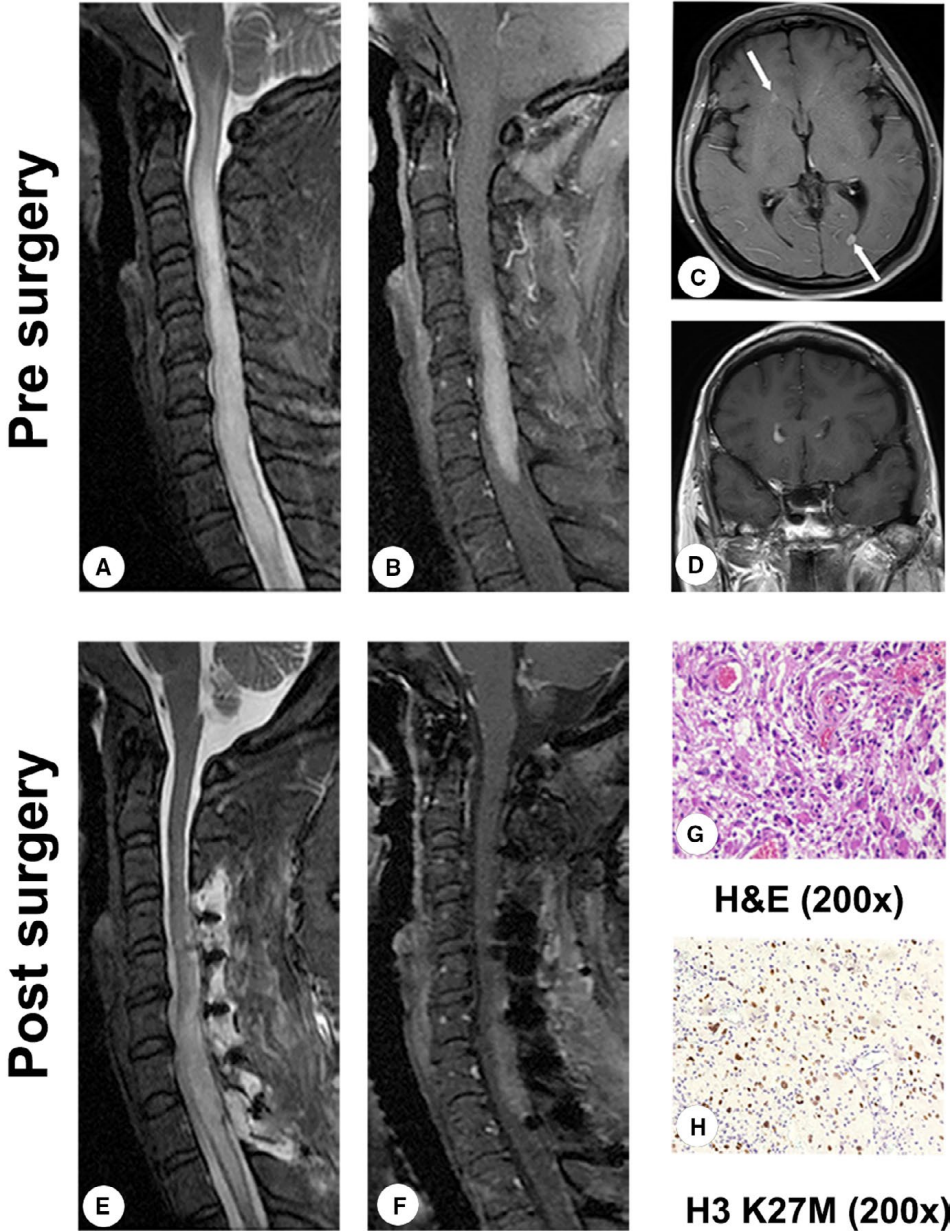
Case 2: Images of an H3 K27M mutation‐positive tumor in a 46‐year‐old female whose main clinical features were dizziness, nausea, vomiting, numbness of the limbs, and weakness of the lower limbs. The histopathology identified an anaplastic astrocytoma. (A, B) Presurgery T2‐weighted and enhanced T1‐weighted magnetic resonance images (MRI). (C, D) Presurgery enhanced T1‐weighted MRI of the brain. (E, F) Postsurgery T2‐weighted and enhanced T1‐weighted MRI. (G) Anaplastic astrocytoma (H&E, 200×). (H) H3 K27M mutation‐positive tumor (H3 K27M, 200×). H&E, hematoxylin and eosin staining

A 46‐year‐old female exhibited dizziness, nausea, vomiting, numbness of the limbs, and weakness of the lower limbs. Presurgery neurological assessment showed grade IV muscle strength in the limbs, increased muscular tension, and sensory decline. The grade of presurgery MMS was 4. Presurgery MRI revealed diffuse spinal cord swelling, T2‐weighted MRI showed a high inhomogeneous signal in the spinal cord at the C4‐T1 level, and enhanced T1‐weighted MRI showed lesions with significant and relatively homogeneous enhancement (Figure 5A,B). Presurgery enhanced T1‐weighted MRI of the brain showed lesions with enhancement (Figure 5C,D). Postsurgery neurological assessment showed grade III‐IV muscle strength in limbs and decreased sensation. T2‐weighted and enhanced T1‐weighted images 1 week after surgery are shown in Figure 5E,F. The histopathology identified an anaplastic astrocytoma, scattered with massive tumor giant cells, with occasional focal necrosis (WHO III‐IV, Figure 5G), the H3 K27M analysis was positive (Figure 5H), and the percentage of Ki‐67‐positive cells was 20%. Although she underwent gross total resection of the spinal cord lesions and received adjuvant radiotherapy after surgery, her survival was only 8.4 months. This observation was consistent with the conclusion of this study that higher WHO grade, higher presurgery MMS, and Ki‐67 expression rate ≥10% were significantly associated with worse survival in high‐grade gliomas.

Previous studies have reported intracranial spread of spinal cord astrocytoma.^24^, ^25^, ^26^ In case 2, the patient had symptoms such as dizziness, nausea, and vomiting before surgery, and the MRI of the brain showed abnormal enhancement of the ventricular wall, so the preoperative intracranial dissemination of the tumor was considered. Due to the development of the disease, the patient did not review the MRI of the brain and cervical spine, and eventually died of coma and dyspnea. The tumor site of the patient was located in the cervical enlargement (C4‐T1), and the recurrence of the original site may lead to limb paralysis without conscious coma. Therefore, we speculate that tumor dissemination may be the most important cause of respiratory center failure and conscious coma.

## 4 DISCUSSION

Due to the rarity of spinal cord astrocytomas, their treatment remains controversial and there are no established special treatment guidelines.^7^ Here, we summarized the clinicopathological characteristics and prognostic factors of spinal cord astrocytomas based on the 2016 WHO classification in 108 patients. Compared with previous studies on diffuse intrinsic pontine gliomas (DIPGs), our study revealed an older age at diagnosis (median, 30 years; range, 4‐63 years), a lower ratio of H3 K27M mutation (38%), and a longer OS (48.1 months) among patients with spinal cord astrocytomas. We also identified that age at diagnosis, WHO grade, presurgery MMS, EOR, and Ki‐67 expression were significantly associated with OS in these patients. Furthermore, a higher EOR was significantly correlated with better survival in patients with low‐grade astrocytomas but not in those with high‐grade astrocytomas. In patients with high‐grade astrocytomas, a higher WHO grade, higher presurgery MMS, and Ki‐67 expression rate of ≥10% were negatively correlated with OS. These findings could provide a basis for evidence‐based practices for managing spinal cord astrocytomas.

In this study, the median age at diagnosis was 30.0 years (range, 4‐63 years). These findings are significantly different from those of previous studies on DIPGs in which the median age at diagnosis was 6‐7 years.^27^ Although histological grade is not a prognostic factor of DIPGs, our study revealed that patients with low‐grade astrocytomas were younger and had longer survival than those with high‐grade astrocytomas.^28^, ^29^ This finding is consistent with previous findings showing that histological grade is an important prognostic factor and that patients with high‐grade (grade III/IV) tumors experienced a worse prognosis.^9^, ^10^, ^11^, ^30^, ^31^, ^32^, ^33^, ^34^ We also identified that the median OS of patients with grade IV tumors was 20.2 months, significantly longer than that reported (<1 year) for patients with DIPGs.^27^, ^35^, ^36^, ^37^ Moreover, the H3 K27M mutation rate was 38%, much lower than the nearly 80% rate in DIPGs.^38^ All these findings indicate that spinal cord astrocytomas are different from DIPGs, and the characteristics of H3 K27M‐mutant gliomas should be considered separately based on their location.

The impact of EOR on the prognosis of spinal cord astrocytomas is different between low‐grade and high‐grade astrocytomas.^11^, ^15^ Ryu et al reported that EOR was not associated with prognosis in patients with high‐grade astrocytomas (total, 26 cases).^11^ A study of 561 low‐grade (WHO grade I/II) astrocytomas indicated that gross total resection could significantly improve survival (HR, 0.22; *P* < .001).^15^ Meanwhile, our study involved all glioma grades and also demonstrated that EOR had a positive effect on survival among patients with low‐grade astrocytomas but not among patients with high‐grade astrocytomas. This finding is inconsistent with previous findings on brain gliomas in which the EOR was not significantly associated with survival in patients with low‐grade (WHO grade II) brain gliomas but showed a statistical significance for high‐grade (WHO grade III/IV) brain gliomas.^39^ Therefore, the treatment for brain gliomas may not be suitable for spinal cord gliomas, and it is urgent to develop a series of standard treatment regimens for spinal cord gliomas.

Consistent with a previous study, the presurgery MMS in this study was significantly higher in high‐grade astrocytomas than in low‐grade tumors.^10^ All patients underwent surgical resection with neurophysiological monitoring and the surgery did not result in significant changes in MMS. The presurgery neurological functional status was associated with OS in all astrocytoma patients or those with high‐grade astrocytomas, but not in the low‐grade astrocytoma subgroup. Thus, surgery should achieve a greater EOR while preserving neurological function.

The chemoradiotherapy regimen for spinal cord gliomas is mainly based on the treatment protocol for brain gliomas. Radiotherapy has a significant positive effect on OS for all patients with brain gliomas, including low‐grade gliomas, while chemotherapy has a significant survival benefit only in patients with glioblastomas.^39^ The efficacy of adjuvant radiotherapy or chemotherapy for spinal cord astrocytoma still remains controversial.^9^, ^15^, ^16^, ^17^, ^18^ In this study, we found that both adjuvant chemo‐ and radiotherapy did not have a significant effect on OS for both low‐grade and high‐grade tumors (all *P* > .05). This result may be related to the low dose of radiotherapy tolerated by the spinal cord and the injury caused by the radiation. Generally, a lower dose (45‐50 Gy) of radiation is administered, and the curative effect is poor.

Although this study involved a large cohort of patients with spinal cord astrocytomas, the sample size is still relatively small, and the power to assess potential predictive characteristics is limited. Aside from having a retrospective design, the study lacked sufficient molecular pathological information. Further prospective studies are needed to address these issues. This study provides data on the characteristics and prognostic factors of spinal cord astrocytomas in the Asian population and has the largest sample of patients with such tumors based on the 2016 WHO classification in a single institution. The findings could be useful for evidence‐based management of spinal cord astrocytomas.

## CONFLICT OF INTEREST

The authors declare that they have no competing interests.

## AUTHOR CONTRIBUTIONS

Yao‐Wu Zhang wrote the manuscript. Rui‐Chao Chai and Yong‐Zhi Wang carefully revised and proofread the manuscript. Yao‐Wu Zhang, Rui‐Chao Chai, and Yong‐Zhi Wang designed the experiment, conducted the information collection, and data analysis. Ren Cao, Wen‐Ju Jiang, and Wei‐Hao Liu collected the clinical information and proofread the manuscript. Yu‐Lun Xu, Jun Yang, and Wen‐Qing Jia conducted the study design and proofread the manuscript. Wen‐Qing Jia and Yong‐Zhi Wang supervised the work.

## ETHICAL STATEMENTS

This retrospective study was approved by the Institutional Review Board of Beijing Tiantan Hospital, Capital Medical University (Beijing, China), and informed consent had been obtained from each patient involved in our study.

## Data Availability

The data sets used and/or analyzed in this study are available from the corresponding author upon reasonable request.
